# Genome-wide chromatin and gene expression profiling during memory formation and maintenance in adult mice

**DOI:** 10.1038/sdata.2016.90

**Published:** 2016-10-11

**Authors:** Tonatiuh Pena Centeno, Orr Shomroni, Magali Hennion, Rashi Halder, Ramon Vidal, Raza-Ur Rahman, Stefan Bonn

**Affiliations:** 1 Research Group for Computational Systems Biology, German Center for Neurodegenerative Diseases (DZNE), Von-Siebold-Straße 3A, 37075, Göttingen, Germany; 2 Research Group for Epigenetic Mechanisms in Dementia, German Center for Neurodegenerative Diseases (DZNE), Von-Siebold-Straße 3A, 37075, Göttingen, Germany

**Keywords:** Epigenetics and behaviour, Fear conditioning, Gene regulatory networks, Sequencing

## Abstract

Recent evidence suggests that the formation and maintenance of memory requires epigenetic changes. In an effort to understand the spatio-temporal extent of learning and memory-related epigenetic changes we have charted genome-wide histone and DNA methylation profiles, in two different brain regions, two cell types, and three time-points, before and after learning. In this data descriptor we provide detailed information on data generation, give insights into the rationale of experiments, highlight necessary steps to assess data quality, offer guidelines for future use of the data and supply ready-to-use code to replicate the analysis results. The data provides a blueprint of the gene regulatory network underlying short- and long-term memory formation and maintenance. This ‘healthy’ gene regulatory network of learning can now be compared to changes in neurological or psychiatric diseases, providing mechanistic insights into brain disorders and highlighting potential therapeutic avenues.

## Background & Summary

Learning and memory processes are crucial for an organism’s capability to adapt to environmental changes. The establishment and maintenance of memory is governed by structural and functional changes of memory-forming neuronal subpopulations. A hallmark of memory-related structural changes is the strengthening or weakening of existing synapses and the formation of new ones, the so-called ‘synaptic plasticity’. These changes are regulated by intracellular signaling cascades that control protein and gene activity^1,2^. Memory-related gene activity changes most probably concur with altered chromatin states, as mutations in chromatin-modifying or chromatin-binding proteins cause learning and memory defects and many neurological and psychiatric diseases^3–6^. These insights are further strengthened by stable and transient memory-related chromatin modification changes in learning genes such as *Reelin*, *Calcineurin*, and *Bdnf*^4,7–10^. So far, studies have primarily used ChIP-qPCR or targeted bisulfite sequencing to scrutinize promoter regions of key memory genes, an approach that is highly sensitive in detecting even small changes but lacks the profound insights into gene-regulatory networks and enhancers that whole-genome techniques could provide. In a nutshell, targeted approaches like ChIP-qPCR yield high precision but very low recall whereas genome-wide approaches like ChIP-seq usually have reduced precision but very good recall.

**Figure 1:**
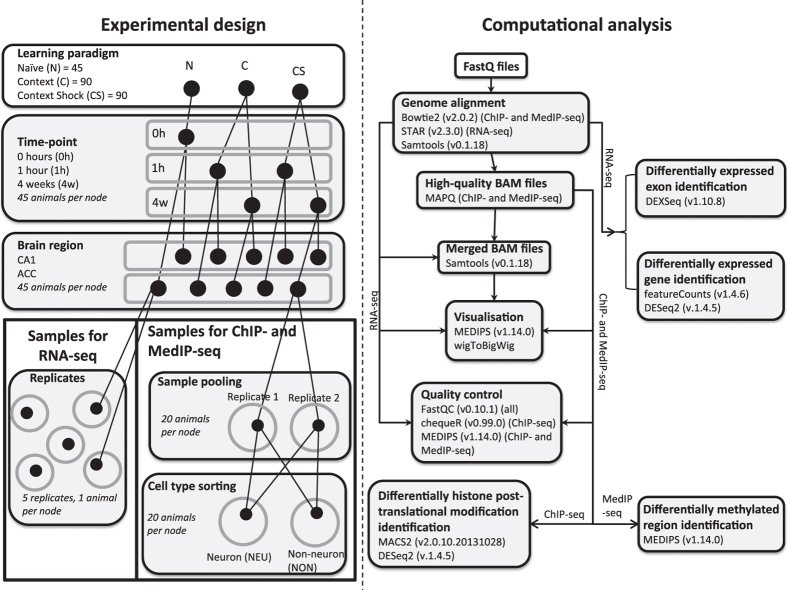
Overview of experimental design and computational analysis workflow. *Left*; experimental design for the generation of samples to be sequenced with ChIP-, MeDIP- and RNA-seq. The mice are trained under different conditions (naïve-N, context-C or context shock-CS) and are sacrificed at different time-points after training (0 h for naïve, 1 h or 4 weeks for context and context-shock). The ACC and CA1 brain regions are then taken from each mouse. For samples that would be used for RNA-sequencing, each tissue sample is used to produce one replicate, resulting in 5 replicates for each combination of condition, time-point and tissue. For samples that would be sequenced with ChIP- and MeDIP-seq, 20 tissue samples are pooled for each tissue to generate two replicates, and the cells within the tissues are sorted by their type (neuronal-NEU or non-neuronal, -NON). *Right*; computational analysis used to process the ChIP-, MeDIP- and RNA-seq data. The main analysis steps involved alignment of the FastQ reads to the mouse genome, visualizing the data, establishing the quality of the data and running differential enrichment/expression analyses; for more information, see ‘Methods’ section.

In an effort to obtain an unbiased, genome-wide view of chromatin modification changes during short- and long-term memory formation and maintenance we performed ChIP-, MeDIP-, and RNA-seq experiments before and after learning, with brain region and cell type-specifically, for several chromatin modifications in three month old male mice (Fig. 1)^11^. As a learning paradigm we chose the very robust and extremely well characterized contextual fear conditioning (CFC) paradigm^12^. We chose to investigate three time-points that correspond to naïve mice (no CFC, 0 h), short-term memory formation (1 h after CFC), and memory maintenance (4 w after CFC). On top of the temporal analysis, two brain areas known to be involved in memory formation (hippocampal CA1 region) and memory maintenance (anterior cingulate cortex, ACC) were investigated^13,14^. From the CA1 and ACC we then extracted nuclei and sorted them into neuronal (NEU) and non-neuronal (NON) populations to very high purity (>95%), using an optimized BiTS-ChIP protocol to gain insights into cell type-specific chromatin modification changes^15,16^. From these cell type-specific nuclei we extracted chromatin for ChIP-seq or DNA for MeDIP-seq experiments using highly-specific, previously validated antibodies^17^.

We restricted the ChIP-seq experiments to the 1 h time-point in the CA1 region, as histone modifications are known to be rather short-lived^9,10,18^. DNA methylation, on the other hand, can be transient or stable and we performed MeDIP-seq analyses in ‘four dimensions’, representing the learning paradigms (naïve—N, context—C, or context shock—CS), the time-point (0 h, 1 h, 4 w), the brain region (CA1, ACC), and the cell type (NEU, NON) (Fig. 1)^7,8,11^. For cell type-specific ChIP- and MeDIP-seq experiments we chose to pool 20 mice per biological replicate since chromatin modification changes were expected to be small, coming from a small population of memory-forming cells. Consequently, the statistical detection of chromatin modification changes required low variance data, which can be obtained by pooling many biological replicates^11^.

Our aim with this data descriptor is to provide the information to easily confirm and extend our analysis results. To this end, we supply raw and intermediate data and ready-to-use bioinformatics analysis code. Interesting future applications could be integrative analyses of the provided data with various published omics and non-omics datasets. To understand if learning-related genes are preferentially epigenetically altered in brain disease, it would be interesting to compare our data to epigenetic changes in various neurodegenerative diseases, such as a recently published data on Alzheimer’s disease (AD)^19^. In order to understand which enzymes are responsible for learning-induced epigenetic changes, published ChIP-seq data of transcription factors and chromatin modifiers in neuronal cell culture and *in vivo* could be analyzed^20,21^. It could also be very informative to link the observed learning-induced epigenetic network changes to genetic information on ‘cognition genes’, highlight commonalities and divergence of memory gene regulatory networks across phyla, and investigate how drugs might affect the signalling pathways that we observed and discuss their potential side-effects^22^.

In summary, this data descriptor provides necessary information, files, and code to reproduce and further analyze learning-related chromatin modification changes, in health and disease.

## Methods

The methods section includes and extends the information of the original manuscript^11^. In addition, references to figures, tables and files of the original manuscript will contain the suffix OM (original manuscript) throughout the rest of this data descriptor.

### Experiment overview and behaviour

A prerequisite for the proposed study is the use of an established, well characterized, and robust learning model. To investigate the molecular mechanisms governing learning, we make use of the very well defined fear-conditioning paradigm. Classical Pavlovian fear conditioning is one of the most commonly used tests for associative memory^12^. In this paradigm mice are trained to associate a novel environment (conditioned stimulus) with an electric foot shock (unconditioned stimulus). The readout for formation of an associative memory is an inborn behavioural response to fear, which is expressed by freezing (no movement). The percentage of the time the animal displays freezing behaviour during context re-exposure correlates with memory strength. This type of contextual fear conditioning (CFC) has been shown to be hippocampus-dependent^13^. Another relevant area is the anterior cingulate cortex (ACC), an area required for long-term memory formation in fear conditioning^14^. Importantly, fear conditioning features high penetrance as a single exposure incurs a robust learning response in mice. Additionally, it has a very well defined time-course of immediate early gene expression, granting insight into the timing of primary and secondary transcriptional responses (Supplementary Fig. 1, OM^11^). Lastly, there is ample evidence that epigenetic changes underlie memory formation during fear conditioning, as published among others by the laboratory of David Sweatt^4^. We have established CFC using a fully automated fear conditioning system (Med Associated) connected to a computer and a control unit containing a shock and a tone generator. Animals are allowed to explore the training cage for 3 min followed by a mild electric shock (2 s, 0.7 mA).

The behavioural analysis was performed with male, 3 month-old mice and only female caretakers conducted the experiments. All mice were acclimatized to housing conditions for 6–7 days prior to behavioural analyses. Naïve, three month-old mice that were not subjected to CFC were used as control (N). Additionally, to separate the associative memory from learning traces associated with the context only, groups of mice were either subjected to the conditioned stimulus only (C) or to the conditioned stimulus and an electric shock (CS).

Mice were sacrificed 1 h (1 h) or 4 weeks (4 w) after CFC. These time points were chosen to reflect short- and long-term memory formation and maintenance, respectively, and are in accordance with published time-courses of immediate early (IE) gene expression after fear conditioning (Supplementary Fig. 23, OM^11^). To study short-term memory formation and the underlying dynamic epigenetic changes we chose one time point within this window, 60 min after CFC. Long-term memory associated epigenetic changes were studied 4 weeks (4 w) after CFC.

Throughout this study, three different cohorts of mice were subjected to CFC:

- **ChIP- and MeDIP-seq cohort**. For cell type-specific ChIP- and MeDIP-seq experiments, tissue from 20 mice for each of the two biological replicates was pooled. We chose to pool this amount of mice per biological replicate since chromatin modification changes were expected to be small given that they come from a small population of memory-forming cells. As a consequence, detection of chromatin modification changes (from a statistical perspective) required low variance data, which can be obtained by pooling many biological replicates. In theory, the variance of the estimator for the true distribution mean *θ* is given by
- $$1/n_{p}*(\sigma_{\varepsilon }^{2}/r_{s}+\sigma_{\xi }^{2}/r_{a}),$$
  where *n*_*p*_ is the total number of pools, $\sigma_{\varepsilon }^{2}$ represents the biological variation, $\sigma_{\xi }^{2}$ signifies the technical variation, *r*_*s*_ denotes the number of individual samples that contribute to a pool, and *r*_*a*_ is the number of sequenced samples for each pool (biological replicates)^23–25^. Given that *r*_*s*_>1 in a pooled design, the concomitant decrease in variance should lead to an increase in power to identify differentially expressed or modified regions.
- In addition, sample pooling reduces the general workload and monetary investment. After subjecting the ChIP- and MeDIP-seq mouse cohort to CFC (see above), the animals were sacrificed 1 h or 4 weeks later and the CA1 and ACC regions were isolated for subsequent nuclear sorting and chromatin and DNA extraction. Whereas MeDIP-seq analyses were conducted for all time-points and regions, ChIP-seq experiments were restricted to cellular consolidation (CA1 1 h). This decision has two main reasons. First, HPTM (histone post-translational modification) changes are reported to occur shortly after CFC (1–3 h) but are rather short-lived^9,10,18^. Second, we did not detect many region-specific changes even during cellular consolidation, which led us to concentrate on the more prominent DNAme (DNA methylation) changes during systems consolidation (ACC 1 h) and memory maintenance (4 w).
- In addition to ChIP- and MeDIP-seq experiments, chromatin from this cohort was also used for immunoblot (IB) analyses as shown in Supplementary Fig. 15 OM^11^.

- **RNA-seq cohort.** For RNA-seq experiments, 5 mice per biological condition were used (5 groups, 1×N, 2×C, 2×CS). In contrast to the ChIP- and MeDIP-seq cohort, the tissue for the RNA-seq experiments was not pooled and not subjected to cell type-specific sorting. In brief, after the RNA-seq cohort was subjected to CFC, the animals were sacrificed either 1 h or 4 weeks later, and the RNA from the CA1 and the ACC regions was extracted. Apart from expression profiling, tissue from the RNA-seq cohort of mice was used for IB analyses as shown in Supplementary Fig. 14 OM^11^.
- **qPCR cohort**. For ChIP-qPCR experiments with high temporal resolution, we used 2 mice per group and 5 groups (1×N, 4×CS). Animals were sacrificed 0, 15, 30 or 45 min after CFC.

### CFC details

Three-month-old male C56BL/6 mice were individually housed in standard conditions with a 12 h light/dark cycle and access to food and water *ad libitum*. All experiments were performed in the accordance with the animal protection law and were approved by the District Government of Niedersachsen (Lower Saxony), Germany. To study learning and memory processes the CFC paradigm was employed with the exception that animals were not subjected to the ‘test’ phase after training. All experiments were conducted in the morning. For training, animals were allowed to explore the context either for 180 s (group: Context—C) or 178 s followed by a 2 s constant 0.7 mA mild foot shock (group: Context Shock—CS) and later were housed back in cages. One group of C and CS (40 animals each) were trained and sacrificed after 1 h, while another group of C and CS (40 animals each) were sacrificed after 4 weeks of training. Animals that did not undergo CFC were used as naïve controls (N, 40 animals). To measure the performance of the learning paradigm, an additional group of mice was trained and tested for memory retrieval 30 min, 24 h, and 4 weeks after context (C) or context shock (CS) exposure (10, 14 and 10 mice per condition, respectively) (see Data Record 1). The motion of mice was tracked and the percentage of freezing was calculated using the Video Freeze automated monitoring system (Med Associates).

### Tissue isolation

Following the behavioural training, the animals were sacrificed by cervical dislocation at the respective time-points (Naïve 0 h, 1 h, 4 w) and whole brain was isolated. Two brain regions implicated in the short- and long-term memory phases of associative learning were dissected and frozen immediately. Throughout the tissue dissection and subsequent sorting samples were kept cold, minimizing processing-related changes in chromatin levels or RNA expression. As depicted in Fig. 1 and Supplementary Fig. 1 of the OM^11^, we chose the CA1 hippocampal sub region to gain information about short-term memory formation and the ACC for long-term memory formation.

To ensure consistent sectioning a single person was in charge of the dissection of the CA1 and ACC while enrichment of region specific marker genes was validated by RT-QPCR. Subsequently, the tissue was dissociated and cell types were sorted.

#### Tissue isolation details

After cervical dislocation the whole brain was isolated in ice-cold Dulbecco‘s Phosphate Buffered Salt (DPBS, PAN-biotech GmbH) supplemented with EDTA-free protease inhibitor cocktail (Roche). The CA1 and ACC regions were isolated under a dissecting microscope using scalpel/needle. Isolated tissues were snap frozen in liquid nitrogen and stored at −80 °C until further processing. In the case of ChIP- and MeDIP-seq experiments dissected CA1 or ACC regions were pooled from 20 mice and stored at −80 °C until further processing.

### Sorting of cell type-specific nuclei

Once the tissues were collected, cell type-specific chromatin for NeuN positive neuronal cells (+) and NeuN negative non-neuronal cells (−) was extracted adapting the BiTS protocol to mouse brain tissue^15,16^. Tissue was thawed in isotonic lysis buffer and immediately subjected to dissociation and fixation. The fixation was performed immediately after cell dissociation in ice-cold buffer and every further step was performed at 4 °C to limit changes in chromatin modifications occurring due to sample preparation to a minimum. Once the single nuclei were extracted and purified through a sucrose cushion, they were stained with NeuN antibody, washed, and stained with Alexa-488 antibody for subsequent fluorescence-activated sorting of nuclei. The pure (>95%) NeuN positive population contains every neuron expressing NeuN endogenously, which are primary excitatory neurons as well as interneurons. NeuN negative cells consist to a very large percentage of glial cells, mostly oligodendrocytes and astrocytes, but also contain other cell types.

The sorted populations of nuclei were then subjected to either DNA extraction or shearing and chromatin extraction. The cell type-specific chromatin was used for ChIP experiments and the cell-type specific genomic DNA for MeDIP experiments.

#### Sorting details

For each replicate, tissues from 20 mice were pooled and nuclei were isolated. All steps except the formaldehyde crosslink were performed at 4 °C or on ice. In brief, frozen mouse tissue from 5 mice was homogenized using a micro-pestle in 500 μl of low sucrose buffer (0.32 M Sucrose, 10 mM HEPES pH 8.0, 5 mM CaCl_2_, 3 mM Mg(CH_3_COO)_2_, 0.1 mM EDTA, 0.1% Triton X-100, 1 mM DTT) and crosslinked with 1% formaldehyde (Sigma Aldrich F1635) for 5 min at room temperature. The reaction was quenched by adding glycine to 125 mM and incubating 5 more minutes. The nuclei were pelleted by centrifugation, re-suspended and homogenized in 3 ml of low sucrose buffer with protease inhibitors (Roche Complete) using a mechanical homogenizer (IKA T 10 basic ULTRA-TURRAX). The nuclei were purified through a sucrose cushion (10 mM HEPES pH 8, 1 M sucrose, 3 mM Mg(CH_3_COO)_2_, 1 mM DTT; 6 ml of cushion for 1,5 ml of lysate) by centrifugation (3,200 rcf for 10 min in Oak Ridge centrifuge tubes), resuspended in PBS, and aggregates were cleared by filtering through a 70 μm filter. The nuclei were stained with anti-NeuN mouse antibody (Millipore mab377) diluted 1:500 in PBS-T, (0,1% Tween 20 in PBS) with 5% BSA and 3% goat serum, incubating for 30 min at 4 °C. The nuclei were washed 4 times with PBS-T and stained for 15 min with anti-mouse Alexa 488 (Life Technologies) diluted 1:1000. The nuclei were washed once with PBS-T and stored in PBS-T with 5% BSA until the sorting. After dissociation by passing the samples through a 26 G needle 10 times, the nuclei were filtered (70 μm) right before sorting on a FACS Aria II (BD Bioscience) into ice-cold conical tubes containing 1 ml of 5% BSA in PBS. The ‘gate settings’ for sorting were based on the size and density of unstained nuclei and both NeuN stained (NeuN+) and unstained (NeuN−) fractions were collected. The average purity of the sorted nuclei exceeded 95%, yielding highly cell type-specific material. After nuclei staining and sorting, a small aliquot was stained with DAPI (1:20.000 in PBS) and mounted on a slide to be imaged using a confocal microscope. A control with unsorted nuclei was also included.

### ChIP-seq

Following the extraction of chromatin, ChIP- or MeDIP-seq experiments were performed. The amount of chromatin available for ChIP and MeDIP experiments is limited as experiments were conducted on very small, cell type-specific, *in vivo* samples, resulting in 5–10 μg (measuring DNA) chromatin for a single biological replicate of 20 pooled mice. Consequently there was an obvious need to establish ChIP and library protocols that give reliable and moreover quantifiable results using limited amounts of material.

In order to accommodate for the limited amounts of chromatin we optimised our ChIP, MeDIP, and library conditions for each chromatin modification individually. The optimized ChIP protocols show reliable enrichment of IP’ed areas with as little as 0.5–1 μg of chromatin as input. We used ChIP-grade antibodies that were previously validated according to the Antibody Validation Database^17^. For the library generation, we have established conditions to generate reliable and moreover quantifiable libraries from as little as 0.5 ng of input material.

To assure that low input libraries do not suffer from high read duplication events due to clonal amplification of reads, libraries prepared with a regular ChIP-seq protocol were compared to libraries prepared with low input amounts. We used the fastQC package to extract sample parameters such as GC ratio, base distribution, and read duplication from raw fastq files. Overall, our low input protocol shows very similar GC ratios, base distribution, and read duplication events, providing strong evidence for unbiased library generation from very low amounts of ChIP-seq input material. Once the samples were single-end 50 bp sequenced on an Illumina HiSeq2000 they were computationally analysed.

It is important to note that we only performed ChIP-seq experiments during the 1 h time point in the CA1, as we could only detect very few region-specific HPTM changes and current literature suggests that HPTM changes might in general be only short-lived^9,10,18^.

#### ChIP-seq details

The sorted nuclei were pelleted by centrifugation at 3,200 rcf for 15 min, transferred to Diagenode shearing tubes and resuspended carefully in RIPA buffer (10 mM Tris-Cl, pH 8.0, 140 mM NaCl, 1 mM EDTA, 1% Triton X-100, 0.1% sodium deoxycholate, 1% SDS and Roche Complete protease inhibitors). The samples were incubated 10 min at 4 °C and then sheared using a Bioruptor Plus (Diagenode; 4 times 5 cycles 30'' ON/OFF High power, spinning down the samples in between). The sheared chromatin was cleared by centrifugation at 16,000 rcf for 5 min, aliquoted in DNA low-binding tubes (Eppendorf), and snap-frozen for storing at −80 °C. The DNA from an aliquot was extracted and purified. The size of the DNA fragments was analysed on an Agilent 2100 Bioanalyzer (Agilent Technologies) using a High Sensitivity chip and the concentration was determined with the Qubit dsDNA HS Assay Kit (Life Technologies). The chromatin was diluted 10 times in IP buffer (50 mM Tris-HCl at pH 8, 150 mM NaCl, 1% NP-40, 0.5% sodium deoxycholate, 20 mM EDTA, Roche Complete protease inhibitors) and pre-cleared with BSA-blocked protein A magnetic beads (Dynabeads, Invitrogen) for 1 h at 4 °C. The appropriate amount of chromatin (as mentioned in Supplementary Table 1 OM^11^) was used for immunoprecipitation by the different antibodies, by overnight incubation on a rotating wheel at 4 °C. Subsequently, 15 μl of BSA-blocked protein A magnetic beads were added to each sample and the mixture was incubated on a rotator at 4 °C for 2 h. The complexes were washed twice with IP buffer with 0.1% SDS, three times with Wash buffer (100 mM Tris-HCl pH 8, 500 mM LiCl, 1% NP-40, 1% sodium deoxycholate, 20 mM EDTA), once more with IP buffer and twice with TE. The beads were resuspended in 1 mM Tris pH 8 containing RNAseA (0.1 μg/μl, Qiagen) and incubated 30 min at 37 °C. The de-crosslinking was performed overnight at 65 °C with 1% SDS and proteinase K (0.5 μgμl^−1^). The supernatant was transferred to a DNA low-binding tube and the beads were washed once more to increase the yield. The DNA was purified by SureClean (Bioline) precipitation in the presence of 15 μg of linear acrylamide (Ambion). The DNA pellet was washed twice with 70% ethanol, dried and re-suspended in Tris 10 mM pH 8. The DNA concentration was determined using Qubit dsDNA HS Assay Kit and the IP efficiency was validated by qPCR on a minimum of 2 negative and 2 positive loci. The sequencing libraries were prepared using either NEBNext Ultra DNA Library Prep Kit for Illumina (New England Biolabs, H3K9ac ChIP) or Diagenode MicroPlex Kit (all other marks) from 1 to 5 ng of DNA after shearing to about 200 bp with 10 cycles (30 s ON, 30 s OFF) on NGS Bioruptor (Diagenode). After adapter ligation, to avoid over-amplification, the number of amplification cycles was determined for each sample by a qPCR on a small aliquot. The libraries were purified by SureClean (Bioline) precipitation and resuspended in 10 mM Tris pH 8. DNA size was determined using a Bioanalyzer chip (DNA high sensitivity) and libraries were validated by qPCR. The sample concentration was measured by Qubit dsDNA HS Assay Kit and adjusted to 2 nM before sequencing (50 bp) on a HiSeq 2000 (Illumina) using TruSeq SR Cluster Kit v3-cBot-HS and TruSeq SBS Kit v3-HS according to the manufacturer’s instructions.

### MeDIP-seq

The optimized MeDIP protocols showed reliable enrichment of IP’ed areas with as little as 0.1 μg of DNA input. For information on the optimisation of MeDIP-seq experiments please refer to the ‘ChIP-seq’ section.

#### MeDIP-seq details

NeuN+ and NeuN- sorted nuclei were centrifuged at 3,200 rcf for 15 min at 4 °C and carefully resuspended in 200 μl of lysis buffer (10 mM Tris-Cl pH 7.5, 10 mM NaCl, 2 mM EDTA, 0.5% SDS, 100 μg Proteinase K) and incubated for 16 h at 65 °C. Genomic DNA was obtained using phenol-chloroform extraction and precipitated by adding 0.3 M sodium acetate pH 5.2 and 400 μl 100% Ethanol. The precipitated DNA was centrifuged at 20,000 rcf for 20 min at room temperature. The pellet was carefully washed once with 70% Ethanol and resuspended in 100 μl of TE buffer (10 mM Tris-Cl, pH 7.5, 1 mM EDTA) with 20 μgml^−1^ RNase A and incubated for 30 min at 37 °C followed by 1 h at 65 °C.

The genomic DNA was sheared using a Bioruptor NGS (Diagenode) for 10 cycles (30 s ON, 30 s OFF) to an average size of 250–300 bp and ~700 ng of the sheared DNA was used further for library preparation. Briefly, the DNA was end-repaired and A-tailed using NEBNext ChIP-Seq Library Prep Master Mix (NEB, E6240 kit) as described in the kit’s protocol. Custom synthesized Illumina paired-end sequencing adaptors (Sigma Aldrich) were ligated as mentioned in the NEB-E6240 kit protocol and 100 ng of the adapter ligated DNA fragments (al-DNA) was used for immunoprecipitation using anti-5-methylcytosine (5 mC) antibody as described earlier for MeDIP^26^.

Briefly, 100 ng of al-DNA was diluted in 42 μl of TE buffer. Additionally, 2 ng of al-DNA was kept aside as ‘input’ sample. The al-DNA was denatured at 99 °C for 10 min and immediately transferred on ice for 10 min followed by addition of 5 μl of 10× IP buffer (100 mM Sodium phosphate, pH 7.0, 1.4 M NaCl, 0.5% Triton X-100), 1 μl of 1:5 diluted 5 mC (Eurogentech, BI-MECY-0100) antibody and 2 μl of protein G Dynabeads (Invitrogen). The immunoprecipitation was followed for 6 h at 4 °C with slow rotation. The beads were captured with a magnetic rack (Invitrogen), supernatant was discarded and washed with 200 μl of 1× IP buffer for three times. The beads were resuspended in 50 μl of proteinase K digestion buffer (50 mM Tris-Cl pH 8.0, 10 mM EDTA, 0.5% SDS) and 3 μl of proteinase K (20 mgml^−1^ stock) was added. The digestion was carried out at 55 °C for 20 min with mild shaking. The beads were captured with magnetic rack and the supernatant was transferred to a microcentrifuge tube. The adapter-ligated and immunoprecipitated DNA fragments (ali-DNA) were further precipitated using linear polyacrylamide (Ambion, AM9520) and SureClean Plus (Bioline, BIO-37048), and finally resuspended in 20 μl of EB (10 mM Tris-Cl, pH 7.5). The MeDIP DNA enrichment was assessed by qPCR. The 2 ng ‘input’ DNA was also made up to 20 μl with EB. The PCR amplification for MeDIP and input samples was followed as mentioned in the NEB-E6240 kit with: 9 μl of DNA sample, 0.4 μl of custom synthesized TruSeq PCR primer cocktail with index-sequence (Sigma Aldrich, 25 mM) and 9.4 μl of 2× Phusion High-Fidelity PCR master mix (NEB-E6240 kit). The DNA was further purified from PCR mix using Agencourt AMPure XP beads (Beckman Coulter, A63880) as per the manufacturer’s protocol. The PCR amplified DNA was quality controlled again for MeDIP enrichment before sequencing.

Finally, MeDIP samples and input DNA libraries were sequenced as described in the ‘ChIP-seq’ section.

### Quantitative PCR (qPCR)

As we could not detect any HPTM changes in the CA1 1 h after CFC, we decided to look at earlier time points studying the immediate early (IE) response to CFC with fine-grained time resolution. We isolated the CA1 and the dentate gyri (DG) regions from 10 mice: 2 naïve mice and 8 mice exposed to context shock as described in the ‘Experiment overview and behaviour’ and ‘Tissue isolation’ sections. 2 mice were sacrificed immediately after CFC (0 min), 2 after 15 min, 2 after 30 min and the last 2 after 45 min. The CA1 regions were processed for ChIP-qPCR for H3K27ac and H3K9ac as previously described but without nuclei sorting and using one mouse per IP. For each mouse, DG from both hemispheres (left and right) were processed separately, one was used for RNA extraction and RT-qPCR and the other one for H3K27ac, H3K9ac and H4K12ac ChIP-qPCR. We checked H4K12ac in addition to the marks used in the rest of the study, as this mark was also described to increase on immediate early genes promoters after CFC^27^. We performed RT-qPCR on 2 IE gene mRNAs that show a strong increase in expression 1 h after context shock exposure.

Furthermore, qPCR was used to assess the specificity of the tissue isolation, as well as to assess the enrichment of ChIP and MeDIP experiments and to validate the integrity of sequencing libraries.

Finally, qPCR was used to validate some of the differentially methylated regions (DMRs) identified by MeDIP-seq analysis. We chose some DMRs induced in the ACC 1 h after CFC and designed primers in the middle of the MeDIP peaks.

#### qPCR details

RNA was isolated using Tri Reagent (Sigma) according to manufacturer’s protocol. RNA was treated with 2 U of DNase I for 20 min at 37 °C, extracted using phenol-chloroform, and resuspended in water before being converted to cDNA using the Transcriptor High Fidelity cDNA synthesis kit (Roche Applied Science). The RT-qPCR was performed on a LightCycler 480 system (Roche) using primers from the Universal Probe Library (Roche) for specific genes. Data was normalized to hypoxanthine guanine phosphoribosyl transferase (*Hprt*) mRNA levels. For MeDIP and ChIP experiments, qPCR was performed using SYBR Green I Master mix (Roche) with custom primers (Sigma Aldrich). For the validation of DMRs, 2 nM MeDIP libraries were diluted 1:50 and 5 μl of DNA was used into 15 μl PCR reaction. The data was analysed using pyQPCR (v0.10dev, pyqpcr.sourceforge.net) with absolute quantification using dilution of inputs as standard samples, error type Gaussian, confidence interval of 90%, maximum E(Ct) of 0.3 and minimum Ct of 35. qPCR quantifications for the ChIP- and MeDIP-seq libraries can be found in Data Record 2.

### RNA-seq

In order to link the changes detected in chromatin modifications after CFC to gene expression, we performed RNA-seq experiments isolating the CA1 and the ACC from a different mouse cohort (see section ‘Experiment overview and behaviour’, RNA-seq mouse cohort). We used 5 naïve mice and 4 groups of 5 mice exposed to CFC. Two groups were only exposed to the context and 2 groups were exposed to the context and received the electric shock (section ‘Experiment overview and behaviour’). Subsequently animals were sacrificed either 1 h (1 h) or 4 weeks (4 w) after CFC. Importantly, the RNA-seq data is not cell type-specific as whole CA1 or ACC regions were subjected to RNA extraction. The data from the naïve samples was used to plot aggregate plots of chromatin modifications for four different gene subsets based on their expression levels (no, low, medium or high expression). Additionally, learning-induced gene expression changes were correlated to DNA methylation changes.

#### RNA-seq details

RNA was isolated as described in ‘Quantitative PCR’ section. Libraries were prepared using the TruSeq RNA Sample Preparation v2 kit (Illumina). The library quality was checked using an Agilent 2100 Bioanalyzer and a Qubit dsDNA HS Assay Kit. Sequencing was performed as described for ChIP samples (section ‘ChIP-seq’).

### Immunoblotting (IB)

For many histone post-translational modifications, predominantly histone acetylation, global increases after CFC were described^10,27–30^. We used IB on whole CA1 regions or cell type-specific chromatin to assess if we could detect these global changes for our set of 6 HPTMs. These two experiments were carried out on different cohorts of mice. Global changes in CA1 regions were investigated in three-month-old male C56BL/6 mice using 5 mice per biological condition (N, C, and CS in the CA1 at 1 h). The IB analyses on cell type-specific chromatin were derived from the previously described chromatin of 20 pooled mice per replicate (sections ‘Experiment overview and behaviour’ to ‘ChIP-seq’).

In this context it should be noted that we could not detect any global changes in HPTMs upon CFC. This could have two reasons, first IB analyses are notoriously insensitive and also unstable, giving rise to variation among replicate samples. Secondly, the CFC protocol that was used is rather mild, consisting of a single 0.7 mA shock. Although the protocol is strong enough to elicit long-lasting behavioural changes in the mouse, it might cause less pronounced global changes in HPTMs. Published global changes are usually induced by stronger CFC protocols, often times using 3 consecutive shocks of 1 mA. The raw IB signals for all comparisons (both whole CA1 regions and cell type-specific chromatin), as well as the ratios calculated per condition, are stored in Data Record 3.

#### Immunoblotting details

Mouse brain regions (CA1 and ACC) were thawed and processed to enrich nuclear proteins. They were homogenized using a micro-pestle in 200 μl TX buffer (50 mM Tris-HCl pH 7.4, 150 mM NaCl, 1 mM EDTA, 1% Nonidet P40, 0.05% SDS) and protease inhibitor (Complete, Roche). After 10 min incubation on a rotating wheel, they were centrifuged for 10 min at 400 rcf. Supernatant was discarded and the pellet was washed with TX buffer (with protease inhibitors) and lysed in TX buffer with 1% SDS by 5 min incubation on a rotating wheel. The samples were then sheared in a Bioruptor (Diagenode) for 15 min (30 s on/off cycle), cleared by centrifugation for 10 min at 9,300 rcf and the supernatant (enriched nuclear proteins) was collected. Protein concentration was measured using Pierce BCA Protein assay kit according to manufacturer instructions in a clear bottom 96 well micro-plate. Absorbance was measured at 562 nm on a TECAN plate reader. After denaturation at 95 °C for 5 min, 4 μg of proteins were run in a pre-casted Bolt Bis-Tris 12% gel (Novex, Life technologies) in reducing condition in MES-SDS buffer (200 V for 35 min). The proteins were transferred to nitrocellulose membrane (pore size 0.2 μm; GE Healthcare) at 50 V for 90 min in cold 1X Tris-glycine transfer buffer with 20% methanol. The membrane was blocked in TBS 0.1% Tween 20 (TBST) with 5% BSA for 1 h (room temperature) and incubated with primary antibody overnight at 4 °C. After 3 washes in TBST, the membrane was incubated with the secondary antibody (IRDyeR 1/10000, LI-COR) for 1 h at room temperature. After 3 additional washes, the membrane was imaged on an Odyssey CLX imaging system (LI-COR). Images were acquired with a resolution of 84 μm and ‘high quality’ settings in 700 and 800 nm channels and the signal was quantified using Image Studio (LI-COR).

Alternatively, IBs were performed using sheared chromatin from sorted nuclei(sections ‘Experiment overview and behaviour’ to ‘ChIP-seq’). The chromatin was diluted in RIPA buffer with 0.1 % SDS and incubated at 99 °C for 10 min. Subsequently, loading buffer was added and the samples were further processed as described for the nuclear protein lysate (see above) using 100 ng of chromatin (measuring DNA) per well.

### Read alignment

The alignment of deep sequencing reads to the mouse genome can be split into three parts, the alignment of ChIP-, MeDIP-, and RNA-seq data.

- ChIP-seq data was aligned to the mouse NCBI genome version 38 using Bowtie2 (ref. 31) (v2.0.2). Reads were first aligned using default parameters allowing for 2 mismatches using seed alignment. In more detail, Bowtie2 (v2.0.2) first searches for end-to-end 0-mismatch alignments *and* end-to-end 1-mismatch alignments and subsequently performs a seed-based alignment with 2 mismatches. For true multi-map reads that align to multiple regions with the same score, only a single alignment was returned. The obtained Sequence Alignment/Map (SAM) files were converted into sorted Binary Alignment/Map (BAM) files using the samtools suite^32^. Subsequently reads were filtered using two alternative options (i) high quality uni- and multi-mapped reads and (ii) good quality uni-mapped reads (see also bowtie2 MAPQ):

- High quality uniquely and multi-mapped reads were obtained by filtering out reads with low quality (MAPQ !={0, 2, 3, 4}).
- Good quality uniquely mapped reads were obtained by filtering out reads with MAPQ scores {0, 1}. This step removes all true multi-map reads (reads that align to several genomic locations with the same score).
- Throughout the manuscript ChIP data was analyzed using high quality uni- and multi-mapped reads (i). In addition, data used to analyse region-specific HPTM changes was also analyzed using good quality uniquely mapped reads (ii). We opted to include high-quality multi-mapped reads in our analyses as the comparison of peak calling and differential HPTM analyses using uni- and multi-mapped reads or only uniquely mapped reads showed very few differences (see also next section for further rationale).

- MeDIP-seq data was aligned and filtered for high-quality reads as described in the ChIP-seq section (see above).
- We would like to emphasize that we specifically opted to consider uniquely mapped and high quality multi-mapped reads in the analysis of MeDIP data for two main reasons. First, high quality multi-mapped reads constitute valid DNA methylated regions and should as such be considered. Second, more than half of DNA methylation reads fall into intergenic regions, many of which are repetitive.

- RNA-seq data was aligned to the genome using gapped alignment as RNA transcripts are subject to splicing and reads might therefore span two distant exons. Reads were aligned to the whole *Mus musculus* mm10 genome using STAR aligner^33^ (2.3.0e_r291) with default options, generating BAM files. Read counts for all genes and all exons (Ensembl annotation v72) were obtained using featureCounts (v1.4.6)^34^.
- For data visualisation, BAM files were converted into WIG and bigWig files using the MEDIPS ‘MEDIPS.exportWIG’ function with a window of 50 bp and RPM normalization. Subsequently, bigWig files were uploaded to our custom-built genome browser, http://memory-epigenome-browser.dzne.de.
- The code used to generate alignment and visualization data can be found in Figshare (https://dx.doi.org/10.6084/m9.figshare.3490613). Raw BAM files (containing all aligned reads) and their corresponding bigWig files, for all ChIP-, MeDIP- and RNA-seq samples, are available as Data Record 4.

### Known cell type-specific gene list

In order to assess the cell type-specificity of the data we manually extracted and annotated a set of neuron and non-neuron specific genes by using publically available data^35,36^ and in-house RNA-seq information. Cell type-specific genes were used for the analyses of cell type-specificity using aggregate gene plots, precision and recall calculations, and finally for the genome-wide prediction of cell type-specific genes.

We would like to state that this list is not exhaustive, as it does not contain all known neuron- and non-neuron-specific genes in the brain. This is also reflected in the prediction of known neuron-specific genes that were not included in our compiled list.

### Aggregate plots

Aggregate plots were used to obtain information on ‘global’ chromatin modification changes on several occasions throughout the manuscript. These profiles depict the average read density per biological condition (e.g., neuronal H3K4me3 in naïve CA1 samples) over defined genomic regions (e.g., genes, *cis*-regulatory modules or CRMs, and intergenic regions). In order to calculate the average signal over multiple regions of different length, each region is fit to a predefined length by either compressing (for long regions) or extending (for short regions) the available read information (spline interpolation). A moving window (MW) of width 5 was used to smooth the average profiles and 1% of extreme values were trimmed on both ends.

In addition to gene plots, we used aggregate plots on CRMs and on intergenic regions. For CRM aggregate plots, we selected all the CRMs that we predicted in the same cell type and tissue. For the intergenic regions, we randomly selected 20,000 regions that were at least 10 kb away from the closest gene. We used aggregate plots to study known cell-type specific genes, global learning-induced HPTM and DNA methylation changes in genes, CRMs, or intergenic regions, on genes classified according to their expression level, and on genes that are differentially expressed upon CFC. In order to further increase our sensitivity in detecting HPTM changes after neuronal activation, we used a similar approach with previously published data on KCl-stimulated neurons in culture.

Aggregate plots for learning- (*in vivo*) (Supplementary Figs 16, 17 and 18 OM^11^) and stimulation-induced (*in vitro*) (Supplementary Figs 19 and 20 OM^11^) global HPTM and DNA methylation changes contain bar graphs that quantify peak HPTM and DNA methylation levels.

#### Plot details

Aggregate gene plots were created using a modified version of ngs.plot^37^ with the parameters -MW 5 (smoothing of average profiles), -RB 0.01 (trimmed mean to remove extreme values), and -L 5000 (flanking 5 kb regions). A 95% confidence interval was included into the aggregate gene plots to estimate sample variance and significance.

To classify genes according to their expression levels, we calculated the RPKM values for the genes of the used RNA-seq data from naïve samples (CA1 and ACC). For non-expressed genes we considered all the genes with zero read counts in all the samples. Genes with an average RPKM between 1 and 5 were considered as low expressed genes, genes with 5 to 30 RPKM were considered as medium expressed genes and genes with RPKM higher than 30 were considered as high expressed genes. In order to visualise learning-related (and stimulus-dependent) coordinated changes in chromatin modifications and gene expression levels, aggregate plot HPTM and DNA methylation signals were subset for differentially expressed genes.

To quantify potential changes, peak chromatin modification levels for each condition were calculated and included as bar graphs into the aggregate plots. Bar graphs were normalised to naïve (N) or unstimulated (Un) chromatin modification levels for *in vivo* or *in vitro* data, respectively.

### Precision and recall calculations

In order to identify whether chromatin modifications can predict cell type-specific gene activity, we calculated the precision and the recall for each chromatin modification based on a set of known neuron and non-neuron specific genes. Precision and recall of each chromatin marker was estimated by measuring the predictive power of our data by comparing the enrichment of a certain mark in all cell type-specific genes. To this end, read counts on transcriptional start sites (H3K4me3 and H3K27ac) or gene bodies (H3K79me3, H3K4me1, H3K27me3, and DNAme) were compared using DEseq2(v1.4.5)^38^. For promoter/TSS specific markers, the reads in a 3,000 bases window around the TSS (−1500 to +1500) were used. For gene body specific markers, the reads for the entire region from the TSS to the transcriptional end site (TES) were used.

Regions with less than 20 reads in total (for all the compared samples) were filtered out and the fitType ‘mean’ was used for DESeq2(v1.4.5)^38^. Precision and recall were calculated according to their usual definitions:

- Precision=TP/(TP+FP)
- Recall=TP/(TP+FN).

True positives (TP), false negatives (FN), and false positives (FP) were calculated based on the observed direction of fold change and the respective known gene annotation (as neuronal or non-neuronal). For active chromatin modifications (H3K4me3, H3K27ac, and H3K79me3), genes with significant (FDR<0.05) positive fold changes (neuronal reads>non-neuronal reads) were annotated as neuronal genes and genes showing significant negative fold changes (neuronal reads<non-neuronal reads) were annotated as non-neuronal genes. Thereby, a significant positive fold change in a known neuron-specific gene was considered a TP. A significant positive fold change in an annotated non-neuron-specific gene was considered a FP. A significant negative fold change in an annotated non-neuron-specific gene, and genes with no significant changes, were considered TN (true negatives) using neuronal data. A similar approach was taken to calculate the precision and recall of non-neuron-specific genes. In the case of repressive chromatin modifications (H3K27me3 and DNAme) the opposite correlation was expected.

It is important to note that the histone modification H3K9ac was not included in precision and recall calculations as it was added to the study relatively late. For the same reason, this modification was also not included in the novel prediction of genes and CRMs.

Finally, the precision and the recall for neurons and non-neuronal cells in CA1 and ACC were calculated using naïve samples that were not exposed to CFC.

### Prediction of novel cell-type specifically expressed genes

Given the predictive power of the individual chromatin modifications, especially of histone modifications, cell-type specific gene expression was predicted genome-wide from naïve neuronal and non-neuronal data of the CA1 and ACC. These predictions are not exclusive, meaning that genes are preferentially expressed in one cell type over the other but are not exclusively expressed in one cell-type. DNA methylation was not used due to its low classification performance and H3K9ac was left out of the analysis because it was included relatively late into the study.

For the comparison of neuronal and non-neuronal data for each chromatin modification a matrix with the read counts for TSSs or gene bodies was created. Subsequently, DEseq2(v1.4.5)^38^ was used to identify statistically significant differential coverage between neuronal and non-neuronal signal. The region around the TSS (−1500, +1500) was used to compare H3K27ac and H3K4me3 ChIP-seq data, and the full gene body was used for H3K79me3, H3K27me3 and H3K4me1.The results were filtered for an FDR<0.05, a |logFC|>1, and a mean coverage >50. For all comparisons neuronal data was considered as treatment and non-neuronal data as control. Consequently, a positive fold change for activity-related modifications indicates increased gene activity in neurons.

To assess cell type-specificity we used a heuristic best-out-of-three classifier. Considering all combinations of chromatin modifications the best-out-of-three classifier performed best on a set of known cell type-specific genes that we compiled. For this classifier, genes have to have three out of five chromatin modifications significantly increasing in order to be predicted as cell-type specific genes. It should be noted that better performance values could most probably be obtained with machine learning classifiers (see section CRM prediction).

To be classified as neuronal gene (meaning more active in neurons than in non-neurons), at least three chromatin modifications have to show statistically significant enrichment (or depletion for H3K27me3) in neuronal data as compared to non-neuronal data. Furthermore, the residual two chromatin modifications should not show enrichment in non-neuronal data. The same procedure was applied to determine non-neuronal active genes. All results were generated using naïve samples that were not subjected to CFC.

In order to understand the biological function of these set of genes, we performed a pathway analysis using IPA.

### Functional gene enrichment analysis

For the identification of enriched functional terms in sets of genes WebGestalt^39^ and QIAGEN’s Ingenuity Pathway Analysis—IPA were used. In general, we only considered gene lists for functional analysis that contained at least 15 different genes.

For WebGestalt, each set of genes was analysed in the WebGestalt web-service by uploading the corresponding gene IDs. GO category enrichment (adjusted *P*-value<0.1) was assessed by calculating the fold-change between the observed and the expected number of genes of a given GO category. Raw WebGestalt files can be examined in the Supplementary Data Set (Files 1–14) OM^11^.

Functional enrichment analyses with IPA ‘Core Analysis’ module were conducted using default parameters. Multi-sample comparisons were conducted using the IPA ‘Comparison Analysis’ module with default settings. The resulting tables for both ‘Canonical Pathways’ and ‘Bio Functions’ were exported into csv files.

The results for each dataset were combined using the ‘Comparative Analysis’ tool from IPA and exported as csv file. Comparative IPA and WebGestalt analyses containing enrichment *P*-values were post-processed using R (heatmap.2 function) to plot hierarchical clustering heatmaps. Heatmaps that display WebGestalt GO term enrichment are limited to GO levels 3 (more general terms) and 6 (more specific terms) to simplify the already complex heatmaps. Although this approach yielded good results in our hands, we would like to stress that in general it is advisable to use functionally- or hierarchically-informed algorithms for GO enrichment, as provided in many forms and shapes in different software suites.

### Integration of published data

To examine changes in histone modifications before and after KCl stimulation of primary neuronal cell culture, published data was downloaded and analysed^21,40^. This experiment elicits a strong depolarisation of almost all analysed neurons, eliminating sensitivity issues arising due to few active cells *in vivo*. This dataset contains ChIP-seq data sequenced with Applied Biosystems’ SOLiD System 3.0 for H3K4me1, H3K4me3, H3K27ac and H3K27me3 from cultured neuronal cells before and after KCl stimulation. SRA files from GEO datasets GSE21161 and GSE60192 were downloaded and converted into csfasta format (fastq-dump). Colour space-encoded data was aligned to the NCBI genome version 38 using Bowtie (v1.1.1) with colour space mapping (-C) and allowing for 1 mismatch in end-to-end alignment (-m 1). Moreover, we used the ChIP-seq enhancer data of the transcription factors CBP, Npas4 and CREB to build the training dataset for the CRM detection.

### CRM prediction

- Active cell type-specific CRMs were predicted using a modified random forest classifier ‘RFECS’^41^ and naïve chromatin modification data. Since RFECS is a supervised machine learner it requires a training dataset to learn the parameters for future classification. Due to the lack of genome-wide CRM, transcription factor binding, and chromatin modification data in murine brain, published primary neuronal cell culture data for key transcription factors (TFs) and chromatin modifications was used^21,40^. The following two sections will first describe the creation of the training dataset and subsequently explain the training, prediction, and validation of the machine learner.
- *Training dataset*. In order to build positive and negative CRM sets for RFECS training (parameter estimation) published cell culture data for TFs (CBP, Npas4 and CREB) and chromatin modifications (H3K4me1, H3K4me3, H3K27ac and H3K27me3) was used.
- A high confidence positive CRM set was created by selecting regions in the mouse genome that contained a peak for either H3K27ac or H3K4me1 and were bound by at least one TF. In more detail, chromatin modification peaks for H3K27ac and H3K4me1 were obtained using MACS2 (peak score exceeding 10) and the peak summits were extended by 1,000 bases in each direction. Subsequently, H3K27ac and H3K4me1 peaks in close genomic proximity (<1000 bases) were merged, obtaining a total of 30,700 chromatin modification-enriched regions. Next, the binding location of each TF was downloaded in bed format and converted to mm10 coordinates (liftOver tool at UCSC website—https://genome.ucsc.edu/cgi-bin/hgLiftOver). Only TF binding sites larger than 200 bp were considered for further analysis and TF binding sites within a distance of 1000 bases were merged. With this information, we selected for enriched chromatin modification regions that were overlapping with TF binding sites by at least one base, resulting in a list of 2101 putative CRMs. We then removed all regions closer than 5,000 bases from any H3K4me3 peak or TSS, obtaining 762 regions that were used as the high-confidence CRMs (positive training dataset).
- In addition to the set of high-confidence CRMs a set of genomic locations that do not represent CRMs was compiled. For this, H3K4me3 peaks (active promoters) and annotated TSS regions (+/− 500 bp around) were combined (115988 regions). From these, all the regions overlapping with a TF binding site were removed (113398 promoter regions). Additionally, random genomic regions that do not overlap with TF binding sites (+/− 5,000 bases) and match the size distribution and number of positive CRMs were selected using bedtools shuffle^42^(random regions). The final negative dataset was created by randomly selecting 10% of the promoter regions and all of the random regions, resulting in a set of 14,879 high-confidence non-CRM regions. Promoter regions are a special case of negative regions in that they are defined by the presence of very high H3K27ac and H3K4me1 modifications, whereas regular negative regions have little to no signal. Since promoter regions are relatively scarce throughout the genome we chose to increase their proportion in the random dataset by adding 10% of known promoter regions. In consequence, the classifier was able efficiently learn negative regions with little to no HPTM signal and very high promoter HPTM signal.

- *Training, prediction and validation*. The machine learner was trained using the sets of positive and negative CRMs (see previous section) and neuronal histone modification data in bed format from the ACC of naïve mice. Given a sample corresponding to a particular tissue and cell type, the genome was divided into windows of 2000 bps, and each window was represented in terms of number of reads spanning over 10 equally-sized sub-windows (200 bps). The subdivision of windows allows the machine learner to consider the amount of reads as well as their shape to determine whether its features correspond better to a CRM or non-CRM region. Using those features and a window size of 2000 bases, a prediction model with 65 trees was built^41^. Predictions were validated by overlapping the predicted sites with H3K27ac peaks and H3K4me1 peaks. Predictions in HK4me3 and TSS regions were considered false positives and were used for sensitivity/specificity calculations. For the final CRM set these regions were manually excluded. At the end the machine learner predicted 60544 unique high-confidence CRMs.

BED files representing the positive and negative regions used to train the RFECS model and the binary file containing the trained forest used for the CRM predictions can be found in Figshare (https://dx.doi.org/10.6084/m9.figshare.3153406). The predicted CRM regions can be found in Data Record 5.

### CRM validation

In order to biologically validate the *cis*-regulatory modules (CRMs) we predicted, we took advantage of a zebrafish reporter assay that allows for a relatively quick measurement of enhancer activity. The Zebrafish Enhancer Detector (ZED) vector contains a sensitive and specific minimal promoter chosen for optimal enhancer activity detection, insulator sequences to shield the minimal promoter from position effects, and a positive control for transgenesis^43^.

#### CRM validation details

The zebrafish wildtype strain AB was used in all experiments. All embryos were kept at 28.5 °C in E3 media (5 mM NaCl, 0.17 mM KCl, 0.33 mM CaCl_2_, 0.33 mM MgSO_4_) supplemented with 10^−5^% methylene blue and were staged according to Kimmel^44^. All experiments were performed in accordance with animal protection standards of the Ludwig-Maximilians University Munich and were approved by the government of Upper Bavaria (Regierung von Oberbayern, Munich, Germany).

To validate enhancer regions, we randomly chose 30 sequences from the 60544 predicted enhancers, 15 sequences from the CA1 and 15 from the ACC. Then, 300 bp regions were selected based on the conservation between mouse and zebrafish. Selected predicted enhancer sequences were synthesized flanked by attL sites and cloned into the pMK-RQ vector (GeneArt). Using LR clonase (Gateway) enhancer sequences were cloned into the ZED vector containing attR sites in front of the gata2a promoter that drives expression of GFP (kind gift of Jose´ Bessa^43^). Successful integration of the enhancer sequence could be confirmed by analytical digest (loss of BglII site).

Zebrafish were injected with 2–4 pl of 25 ngμl^−1^ plasmid DNA of ZED vector containing the respective enhancer sequence to be validated at the 1 cell stage. Injected eggs and controls were cultured at 28 °C in E3 buffer until analysis. Injected larvae were analysed for transient expression of the dsRed reporter in somites and GFP expression driven by the cloned enhancer element at 2 and 5 days post fertilization (dpf). Larvae were anesthetized with Tricaine (0.016% w/v) and mounted in 3% methylcellulose on coverslips. Fluorescent images were taken using an LSM710 META inverted confocal microscope (Zeiss) and assembled in Photoshop 8.0 (Adobe Systems). GFP-positive cells that had a clear neuronal shape or were located in the CNS were scored as neuronal. The enhancer element was scored as non-neuronal if GFP was expressed in non-neuronal cells in at least 20 injected embryos.

### Genomic annotation of CRMs and DMRs

CRMs were associated with their surrounding genomic context in order to understand their possible functional role. This genomic context can be either a gene (the region is located inside a gene), the closest gene (the region is next to a gene), or intergenic (the region is not next to a gene). Given a CRM resides inside of a gene, the gene substructure can be annotated, such as 5′-UTR, exon, intron, 3′-UTR. To assess if CRMs preferentially reside in specific genomic regions we used a permutation-based test to calculate significance. In brief, we simulated random regions with the same number and size of the CRMs and assessed the enrichment or de-richment of specific genomic features.

Similarly, we needed to annotate DMRs with the genes they potentially affect. To this end, DMRs inside a gene, in the promoter region of a gene (1000 bp upstream) or in the terminator region of the gene (300 bp downstream the TES) were considered gene-associated DMRs or, in other words, differentially methylated genes (DMGs). For genes containing multiple DMRs only one DMG entry is considered. For DMGs containing hyper- and hypo-methylated DMRs one hyper- and one hypo-methylated DMG is considered. This last relation is important since it explains why the number of hyper- and hypo-methylated DMGs is always equal to or bigger than the total number of DMGs. DMG information was then used for gene enrichment analyses using WebGestalt^39^ or IPA.

#### Annotation details

The annotation of CRMs and DMRs with genomic regions was performed using the bedtools intersection function comparing the CRM or DMR locations in bed format to a genome annotation bed file^42^. The mouse genome annotation bed file was extracted from UCSC tables (http://genome.ucsc.edu/cgi-bin/hgTables) using the Ensembl mm10 genome annotation. Individual bed files were downloaded for whole transcripts (from TSS to TES), exons, introns, 3′-UTRs, and 5′-UTRs. As a first approximation, CRMs were annotated as intra- or intergenic. Intragenic regions are defined as overlapping with annotated genes including a promoter region (−1000 to 0 bases from the TSS). Intragenic regions were further annotated for overlap with genic features such as the promoter (−1,000 to 0 bases from the TSS), 5′-UTR, exon, intron, or 3′-UTR. Intergenic regions were annotated to the closest gene and the distance was recorded.

For the enrichment analysis the set of results was shuffled randomly using the ‘bedtools shuffle’ function over the entire genome^42^. This process was repeated for 10,000 iterations and the significance was estimated using the fraction of random associations with a higher overlap as compared to the true overlap (e.g., after 10,000 permutations the overlap of random CRMs with exons is higher than the observed true overlap in five instances, giving rise to a *P*-value of 0.0005).

### CRM motif enrichment

Homer (v.4.6) (http://homer.salk.edu/homer/) was used for the discovery of enriched motifs. BED files containing the CRM positions (provided in Data Record 5) were submitted to the findMotifsGenome.pl script using the parameters -size 500 -len 8 and -mask in order to mask repetitive regions and avoid too many false positives. Neuronal and non-neuronal CRMs were compared based on their motif enrichment. TFs for the enriched motifs were manually merged according to their similarity and only TFs expressed in brain tissue (based on our RNA-seq data) were selected.

### Region-specific HPTM changes

To analyse learning-related region-specific HPTM changes read counts on transcriptional start sites (H3K4me3 and H3K9ac), gene bodies (H3K79me3 and H3K27me3) or called peaks (H3K4me1 and H3K27ac) were compared. For promoter/TSS specific modifications, the reads in a 1,500 bases window around the TSS (−500 to +1000) were used. For gene body specific markers, the reads for the entire region from the TSS to the TES were used. For HPTMs that do not reside at predefined genomic positions, such as H3K27ac and H3K4me1, MACS2 (ref. 45) (v.2.0.10.20131028 tag: beta) was used to call peaks from merged BAM files, using parameters shiftsize=FS/2 and q=0.01, where FS is the fragment size estimated by the in-house ‘chequeR’ package (https://dx.doi.org/10.6084/m9.figshare.3487679), which can estimate fragment sizes based on uniquely mappable regions and genomically informative regions (e.g., transcription start site regions).

DEseq2 (v1.4.5)^38^ was applied to identify the differential coverage comparing naïve to context only (N-C), naïve to context shock (N-CS), or context to context shock (C-CS) samples of all annotated mouse Ensembl genes and MACS2 peaks. Regions were required to contain at least 2 reads per 50 bp in a given TSS, gene body or MACS2 peak when averaging over samples of each condition. Due to the reduction in the number of genes/peaks that have to be statistically evaluated, the multiple-testing burden is decreased, resulting in lower *P*-values for the analysed genes. Lastly, we only considered HPTM changes with an adjusted *P*-value<0.1 and we did not apply any fold-change thresholds. These settings are rather lenient allowing for the detection of small changes while not detecting too much noise due to low coverage variation. It is important to note again that small changes were expected as only a fraction of the cells analysed would partake in the formation of memory (see also main text, OM^11^).

Differential HPTMs (DHPTMs) for called peaks (H3K4me1 and H3K27ac) were associated with their surrounding genomic context. Genes containing DHPTMs up to 5 kbp upstream the TSS were used for functional analysis.

Genes containing DHPTMs were compared to the differentially expressed genes (DEGs) (for the calculation of DEGs please refer to the section ‘Differential expression of genes and exons’) and the overlap significance was calculated using a two-sided Fischer’s Exact Test. DHPTM-DEGs gene lists that contain more than 15 gene entries were analysed for GO enrichment.

The peak caller MACS2 detects rather small regions and might therefore not be the best software to analyse larger regulatory genomic regions (*cis*-regulatory regions as opposed to *cis*-regulatory modules). Consequently, H3K27ac peak calling for *cis*-regulatory regions was performed using RSEG^46^ (v0.4.4) that was specifically developed for broad, dispersed peaks. To run RSEG we converted the H3K27ac BAM files into bed files using bedtools^42^. In addition to the output and input files we used the following RSEG parameters: -i 20 (default number of iterations for RSEG internal HMM training) and -c mm10.bed (list of chromosome sizes for mouse NCBI genome version 38). The code used to identify DHPTMs can be found in Figshare (https://dx.doi.org/10.6084/m9.figshare.3490613) while the actual DHPTM regions can be found in Data Record 6.

### Region-specific DNA methylation changes

For the analysis of DMRs the R package MEDIPS^47^ was used. This package contains many functions to evaluate the quality of the data (MEDIPS.correlation and MEDIPS.saturation were used) and to identify DMRs between two different conditions. MEDIPS identifies DMRs by binning the genome with a fixed window size and identifying positions with significantly different number of reads.

In order to obtain optimal results, two MEDIPS parameters need to be adjusted: the window size and the minimum number of reads per window. Whereas big windows generate broad methylated regions by increasing the chance of merging neighbouring peaks, small windows might fail to identify large methylated regions. Thus, whereas small windows increase resolution they carry the heavy burden of a large multiple-testing adjustment, as MEDIPS tests every genomic region for differential enrichment. Similarly, only regions with a reasonable number of reads should be analysed to alleviate the multiple-testing burden.

When optimising the window size and read number for all possible C-CS comparisons we were unable to detect a single best set of values that would optimize the DMR number for all comparisons. Therefore, we selected average values that performed best overall conditions. Moreover, the MEDIPS parameter 'uniq' was set to ‘true’ in order to discard PCR duplicates.

Finally and importantly, we first thought of using a false-discovery rate (FDR) of 0.1 for all comparisons in order to detect small methylation changes, as only a subset of cells was expected to represent a network correlate of memory and therefore change their methylation. Surprisingly, the number of DMRs in N-C and N-CS comparisons was high so that we decided to reduce the *P*-value to 0.05 for these comparisons. Consequently, DMRs for N-C and N-CS comparisons have an adjusted *P*-value<0.05 whereas C-CS DMRs have an adjusted *P*-value<0.1.

#### DMR details

For MeDIP-seq analyses, the MEDIPS^47^ package (v1.16.0) was used. The optimization of the window size and the minimum number of reads was performed for windows of size 100 bases up to 3,000 bases using 100 bases increments. For each window size, minimum read numbers ranging from 1% of the window length to 15% of the window length were tested (e.g., for window size 100 and a 1% minimum read number at least 1 read has to be found). It was noticed that the optimum curve shows notable changes in number of DMRs depending on the analysed sample (data not shown). We found the optimum (highest number of significant DMRs) window size between 500 and 800 bases with read filtering values around 4–6%. A window size of 700 bases and 5% minimum read filtering were chosen for all comparisons. Reads were extended by 250 bases ('extend'=250). DMRs were filtered by FDR<0.05 in all N-C and N-CS comparisons and by FDR <0.1 in all C-CS comparisons. DMRs with positive fold changes were called hyper-methylated and DMRs with negative fold changes were called hypo-methylated. In N-C and N-CS comparisons naïve samples and in C-CS comparisons context only samples were used as control samples. The code used to identify DMRs can be found in Figshare (https://dx.doi.org/10.6084/m9.figshare.3490613),while the actual DMRs regions can be found in Data Record 7.

### Differential expression of genes and exons

The processing and quality control of the RNA-seq data were performed as described in the section ‘Quality control details’. Read counts were generated using featureCounts (v1.4.6)^34^ and naïve (N), context only (C), and context shock (CS) samples were compared using DESeq2 (v1.4.5)^38^. Genes with a *P*-value<0.05 were considered to be differentially expressed. It is noteworthy that one of the 5 CA1 samples from the CS1 h group was excluded from the analysis as it was considered an outlier.

For the analysis of differential exon usage, read counts for each exon (Ensembl annotation GRCh38.74) were generated using featureCounts (v1.4.6)^34^ and naïve (N), context only (C), and context shock (CS) samples were compared using DEXSeq (v1.10.8)^48^. Exons with a *P*-value<0.1 were considered to be differentially expressed.

The code used to do differential expression analyses of genes and exons can be found in Figshare (https://dx.doi.org/10.6084/m9.figshare.3490613). The DEGs and DEEs can be found in Data Record 8.

### DMR gene expression comparison

The differential methylation of promoter or genic regions can alter the expression and the splicing of genes, respectively^49,50^. In order to assess if the detected learning-related DMRs could functionally alter gene expression, the differential expression of genes (DEG) and their exon expression (DEE) was analysed using RNA-seq data. Differentially expressed genes with *P*-values<0.05 and differentially expressed exons with *P*-values<0.1 were considered for DMG gene/exon expression comparisons. Moreover, we included published gene expression changes during systems consolidation (C-CS, ACC 1 h) in our analyses^51^.

Finally, DEGs and DEEs were overlapped with the DMGs and the statistical enrichment of their association was assessed using a right-tailed Fisher’s Exact Test. The functional analysis (WebGestalt GO enrichment) of the overlap between DMGs and DEGs or DMGs and DEEs was combined in a hierarchically clustered heatmap.

### Code availability

We provide code to re-analyse the data in the Figshare repository https://www.figshare.com under the digital object identifiers (DOIs) specified in Table 1. Code is written in shell and is organized into unix-style command-line scripts that perform certain logical steps of the analysis (such as QC and detection of ChIP-seq peaks) and can be run on Linux and Mac operating systems. The PDF tutorial run_scientific_data_analysis.pdf explains how the scripts can be used to analyse parts or all of the supplied data and each script has a help function that explains its usage. Since code is supplied in an interpreted language (shell), users can extend or change the functionality of the tools by simply editing the scripts, allowing for easy customization. The analyses can be run on any computer containing at least 16 Gb of RAM, which should include recent desktop and laptop computers. Whereas processing power is most probably not a limiting factor with most recent CPUs, hard disc space is given that BAM files for all data supplied will require ~255 Gb of disc space. Due to these requirements, we recommend the users to run the analyses sequentially for sample subsets.

In the figshare repository you will find a tutorial run_scientific_data_analysis.pdf explaining how to run all scripts for example ChIP-, MeDIP- and RNA-seq data. The demo data mentioned in the tutorial was used to run the entire analysis on a Mac x64 computer with 16 Gb RAM. All analyses managed to run without errors and generate the expected results, with the maximum memory consumption being 13.5 Gb for the conversion of merged BAM files into bigWig files, and the maximum runtime being more than 7.5 h for the DEE analysis.

## Data Records

All data records are available to be downloaded from Figshare, where Data Records 4, 5 and 7 were deposited and released with the original publication by Halder *et al.*^11^.

### Data Record 1

Excel sheet with freezing percentages for mice trained under conditions context (C) or context-shock (CS) and tested 30 min (30 min), 24 h (24 h) or 4 weeks (4 w) after training (Data Citation 1).

### Data Record 2

qPCR data in XML (can be opened with pyQPCR v0.10.0) and Excel files containing raw expression levels of particular genes. Four of the files (20130426-CA1-1h-K27ac-K79me3-K4me3-H3-lib_qPCR, 20130715-ACCnaiveK79me3K27ac_qPCR, 20131125-CA1-Naive_qPCR, 20150122-CA1-K9ac_qPCR) have been run for various ChIP-seq samples to validate the quality of their libraries, and two files (20150729-MeDIP-LIB-ACC_qPCR, 20150802-ACC-lib_qPCR) were utilised for validation of several DMGs using samples from ACC tissue in conditions naïve, context and context-shock 1 h or 4 weeks after conditioning (Data Citation 2).

### Data Record 3

Excel file contains immunoblotting signals and ratios across conditions Naive (N), context at 1 h (C) and context-shock at 1 h (CS) for chromatin markers H3K9ac, H3K4me3, H3K27ac, H3K27me3 and H4K12ac in ACC and CA1; in addition, the file contains signals and ratios of CA1 neuronal H3K9ac (Data Citation 3). Each table contains the tested sample names (based on their condition), chromatin mark, immunoblot channel, number ID and signal strength, total, area and background. Each replicate had its signals compared between the chromatin mark and H4 signal immunoblotting, resulting in the ratio signal and standard deviation between the signals. The average signal for each condition N, C and CS and standard deviation of said signals were also recorded, and finally, some immunoblots run twice for the same chromatin mark had their averages and standard deviations calculated together.

### Data Record 4

ChIP-, MeDIP- and RNA-seq data are available in SRA format (easily converted to BAM with the SRA toolkit) under accession numbers GSE74964 (Data Citation 4), GSE74965 (Data Citation 5) and GSE74966 (Data Citation 6), respectively. ChIP- and MeDIP-seq data is also available in bigWig format at GEO on the aforementioned locations. RNA-seq bigWig files have not been stored in GEO as their statistical analysis is more valuable than their visualisation (see Data Record 8).

All samples can be visualized in our genome browser http://memory-epigenome-browser.dzne.de (Select Tracks→CHIP-DATA, MEDIP-DATA or RNASEQ-DATA) and downloaded from there in bigWig format.

### Data Record 5

Predicted cell-type- and tissue-specific CRMs are available for visualization on our genome browser (Select Tracks→ENHANCER-DATA; 4 files) and can be downloaded in GFF format therein. The results are also available as an Excel file in Figshare (Data Citation 7), containing all predicted CRMs for each cell-type (NonNeuron or Neuron) and tissue (ACC or CA1) combination. Each table includes the CRM coordinates and the RFECS probabilities for each CRM (for more information, see section ‘CRM prediction’).

### Data Record 6

Excel file containing raw results of the DHPTM analyses between different training conditions on tissue-, cell-type-, time-point- and chromatin-mark-specific samples is available in Figshare (Data Citation 8). The tables contain columns for specifying region location (chromosome name, start and end positions and strand), base mean over tested samples, log2 fold changes between conditions, and statistics of interest (i.e., *P*-values and Benjamini-Hochberg adjusted *P*-values).

### Data Record 7

Tissue-, cell-type- and time-point-specific DMRs between conditions are available for visualization in the genome browser (Select Tracks→DMR-DATA; 19 files) and can be downloaded in GFF format. The raw DMR results are available to download as an Excel file from Figshare (Data Citation 9), where each table contains region coordinates (chromosome name, start and end positions), log2 fold change, *P*-value and adjusted *P*-value for each methylated region in each test. Tables that do not contain the suffixes ‘_raw’ have annotated DMRs for adjusted *P*-value 0.1 in C-CS tests or for adjusted *P*-value 0.05 in N-C and N-CS tests. Those tables contain, in addition to the columns in the raw results, the gene name, type (protein coding, antisense, micro RNA and so on), ENSEMBL transcript ID and gene structure (intronic, 5UTR, downstream and so on) based on the nearest gene to each DMR.

### Data Record 8

Excel file containing raw results of the DEG and DEE analyses between different training conditions on tissue- and time-point-specific samples is available in Figshare (Data Citation 10). This data is also available as part of the GEO dataset GSE74966 (Data Citation 6). For the DEGs, the tables contain the gene’s ENSEMBL ID, name and description, along with chromosome name, start and end positions, strand, gene type (protein coding, micro RNA, long non-coding RNA, and so on), base mean over tested samples, log2 fold change, *P*-values and Benjamini-Hochberg adjusted *P*-values. For the DEEs, the tables contain columns for each exon with their associated ENSEMBL gene ID, exon number, *P*-value, adjusted *P*-value, base mean and log2 fold change.

## Technical Validation

The following paragraphs summarize some of our best practices in experiment planning and technical validation.

### Experimental design

Maybe the most crucial step in a research project is the study design. This is almost always a balance between expected biological and technical variation within groups versus the money and amount of specimen available. Given unlimited biological material and money the choice is simple, the more the better because statistical inferences become more robust. Unfortunately, data and resources are limited so one needs to rely on statistical approaches on how to choose an ‘optimal’ experimental design. However, how do you validate statistical considerations such as sample size?

**Figure 2:**
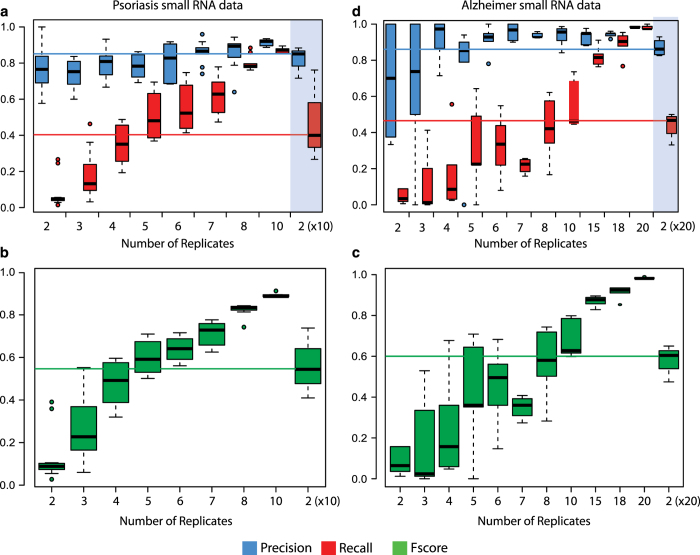
Statistical power of pooling samples. SmallRNA sequenced psoriasis and Alzheimer samples (Data Citation 11 and Data Citation 12, respectively) were run for differential expression (DE) analyses; each dataset consists of wild-type and treatment samples. Both DE analyses (**a**,**b** Psoriasis and c-d Alzheimer) were run using different numbers of randomly selected control and treatment replicates (white background). Alternatively, all samples for each test were pooled into two replicates for each condition, resulting in two control replicates and two treatment replicates from both datasets. Each analysis was run 5 times, and the resulting precision/recall (**a**,**c**) and F score (**b**,**d**) values were used to generate the bar plots. The bar plots appearing in opaque blue represent the results from the analyses run on pooled samples, with the horizontal lines drawn at the mean values to allow comparison between the pooled results and replicate subsampling results.

In our specific case we had to estimate what expected benefit pooling would yield. Although the mathematical model of pooling was described in the section ‘Experiment overview and behaviour’, we went a step further and validated the statistical considerations with a pooling simulation for count data and the effect it has on the calling of differentially occupied/expressed genomic loci as shown in Fig. 2.

For this analysis we used small RNA-seq data given that several studies exist in the literature that analyse over 20 biological replicates; in contrast, we were unable to find a single published ChIP- or MeDIP-seq dataset with more than three biological replicates. In brief, small-RNA sequencing from psoriasis and Alzheimer data (Data Citation 11 and Data Citation 12, respectively) where subsampled with different numbers of replicates for both control and treatment (Fig. 2). Boxplots with white background show precision (blue), recall (red), and F-scores (green) for single replicates randomly chosen from the total set of replicates. Boxplots with blue background show precision, recall, and F-scores for in silico pooled samples. In order to pool samples in silico, reads of true biological replicates were sampled randomly and merged into a single pooled sample. The total number of randomly sampled reads for the pooled sample was equal to the average number of reads from the single replicate input samples. This resulted in the pooling of 5 individual replicates per biological pooled replicate for the Psoriasis data and 20 (10) for the Alzheimer data (20 for the AD and 10 for the control cases).

For the psoriasis data, the precision/recall (a) and F-score (b) for 2 pooled replicates is comparable to that of 5–6 single replicates. For the Alzheimer data, precision/recall (c) and F-score (d) for 2 pooled replicates is comparable to 6–8 single replicates. These simulation results strongly suggest that pooling of samples reduces the variability and increases the power to detect statistically significant changes in count-based NGS experiments, further validating our statistical considerations and experimental setup. We believe that this kind of analysis is simple to perform and can be a very helpful technical validation of study design.

### Behavioural experiments

**Figure 3:**
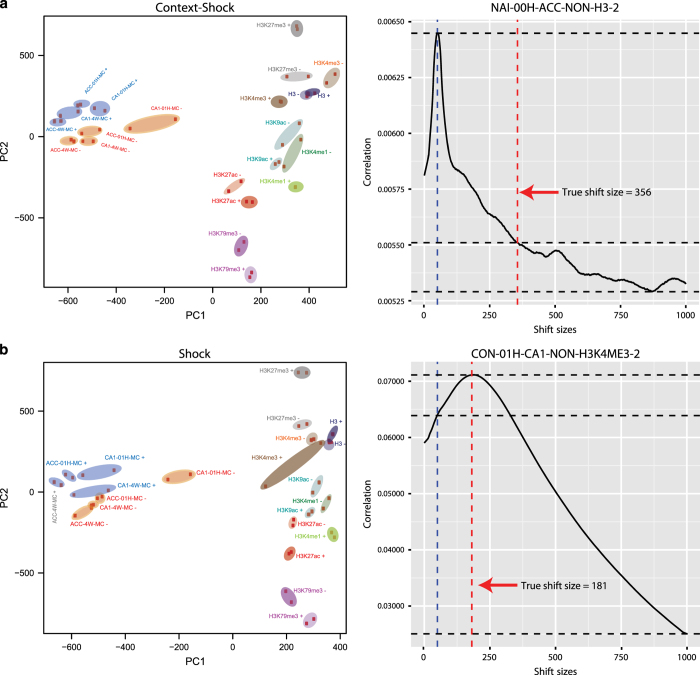
Quality of ChIP- and MeDIP-seq samples. (**a**) Principal component analysis (PCA) plots of context and context-shock ChIP- and MeDIP-seq samples. The plots show the clusters of all replicates with the same conditions, where the MeDIP-seq samples (appearing on the left sides of the plots) are very distinguishable from the ChIP-seq samples (appearing on the right sides of the plots). (**b**) Plots for fragment size estimation, showing the cross-correlation between reads in the positive and negative strands for read shifts 0–1000. Left; plot for ChIP-seq naïve ACC non-neuronal sample targeted by H3 histone marker, showing the low enrichment of a sample with highest cross-correlation at the read length. Right; plot for ChIP-seq context after 1 h CA1 non-neuronal sample targeted by H3K4me3 histone marker, showing a high enrichment of a sample with highest cross-correlation after the read length.

All analysis results and interpretations rely on the assumption that mice that were exposed to the learning paradigm did actually form a memory. We should remark that this is only an assumption, given that individual mice, just as humans, tend to behave and learn quite differently. In order to confirm that the chosen learning paradigm (CFC) induces a robust and long-lasting memory, 40 mice were tested for freezing behaviour 4 weeks after CFC exposure (Figs 1 and Fig. 3). Mice that were only exposed to the novel environment (C) should show no increased freezing, whereas mice that were exposed to the novel environment paired with a shock (CS) should show increased freezing behaviour. In all tested cases, the CFC protocol resulted in robust learning, as all CS-exposed mice showed increased freezing behaviour 4 weeks after learning.

### Tissue-and cell type-specificity

To validate that the brain regions that were dissected really corresponded to the hippocampal CA1 and the cortical ACC regions we performed qPCR on region specific marker genes, specifically of Lphn2 (CA1), Bok (CA3) and Hkdc1 (ACC). Given that CA3 and CA1 are in close proximity and their tissues may have been accidentally mixed, the CA3 region was used as a negative PCR control to verify the purity of the CA1 region. Average fold-change values for six mice showed clear specificity for all regions, as shown in Supplementary Fig. 4 OM^11^.

The purity of cell type-specific sorting (BiTS-ChIP/-MeDIP protocol) was confirmed by re-sorting of samples and visual inspection under a microscope of stained specimen. In all cases a cell type-specificity >95% was required for further analysis.

### Validation of deep sequencing data

Arguably the second most important step is to assess the quality and validity of the raw data. This is especially true for deep sequencing approaches that involve many processing steps at different levels. This paragraph will highlight the steps that we take on a routine basis to assess our sequenced datasets. As a first step, ChIP-, MeDIP-, and RNA-seq data was subjected to an in-house quality control workflow. In brief, sequencing data was analysed for general read quality, genome alignment, average per base coverage, read saturation, replicate correlation, enrichment, and was visually inspected in a genome browser at http://memory-epigenome-browser.dzne.de. Only data passing all quality standards was used for further analyses. Table 2 and Table 3 show the overall quality of the data in terms of alignment quality (Table 2) and unique or multi-mapping of reads (Table 3).

In detail, read quality was assessed to identify sequencing cycles with low average quality, adapter contamination, or repetitive sequences from PCR amplification. Read duplication events were analysed to exclude samples with high PCR bias. The percentage of high quality aligned and uniquely aligned reads was used to estimate possible sequencing biases or contamination. Furthermore, we assessed if samples were sequenced deep enough (number of total reads) by analysing the average per base coverage and the saturation correlation for all samples. To estimate the variance between biological replicates the Pearson correlation was calculated. Principle component analysis (PCA) was performed to assure that samples cluster into their respective biological groups (e.g., for chromatin modification, cell type, brain region) (Supplementary Fig. 3, OM^11^). ChIP-seq peak enrichment was assessed by calculating normalized strand cross correlation (NSC) and relative strand cross correlation (RSC) coefficients using the in-house R package ‘chequeR’. In general, good quality libraries have an *NSC>1.05* and an *RSC>0.8* although these values also depend on the analysed modification or histone^52^. Samples were also inspected visually in our custom genome browser at http://memory-epigenome-browser.dzne.de. Finally, ChIP and MeDIP (differential) enrichment was assessed by qPCR to validate the integrity of sequencing libraries and corroborate statistically significant differences. The data was analysed using pyQPCR (section ‘Quantitative PCR’) with absolute quantification using dilution of inputs as standard samples. An overview of the general data quality can be found in Supplementary Table 3 OM^11^, containing read quality, alignment, saturation, correlation, and enrichment information.

#### Quality control details

Read quality was assessed using fastqc^53^ (v0.10.1) and alignment was analysed using samtools flagstat^32^ (v0.1.18) with default parameters. To calculate the saturation and correlation of ChIP-seq and MeDIP-seq samples the MEDIPS R package^47^ was used. The saturation function splits each library in fractions of the initial number of reads (10 subsets of equal size) and plots the convergence. The correlation between biological replicates was evaluated using Pearson correlation (function MEDIPS.correlation). By using MEDIPS R objects for all samples, the principal components were plotted based on the read density distribution of each sample. This analysis was conducted using the PCA function of the FactoMineR R package. The NSC and RSC enrichment tests were applied to all ChIP-seq samples using the in-house R package ‘chequeR’.

The code used to establish the quality of the data can be found in Figshare (https://dx.doi.org/10.6084/m9.figshare.3490613).

## Additional Information

**How to cite this article:** Centeno, T. P. *et al.* Genome-wide chromatin and gene expression profiling during memory formation and maintenance in adult mice. *Sci. Data* 3:160090 doi: 10.1038/sdata.2016.90 (2016).

## Supplementary Material



## Figures and Tables

**Table 1:** Figshare references to the code used to generate the data records of this data descriptor.

**Figshare repository**	**Data descriptor section**	**DOI (digital object identifier)**
Scripts	Scripts used to run the various analyses, including the manual exaplaining how to run them	DOI:10.6084/m9.figshare.3490613
Demo	Example samples used to show how to run the analysis	DOI:10.6084/m9.figshare.3487391
References	Reference files for mm10 used by the analysis scripts	DOI:10.6084/m9.figshare.3487679
CRMs	CRM prediction	DOI:10.6084/m9.figshare.3153406
The table includes the name of the Figshare repository, the section it refers to in this data descriptor and their digital object identifiers (DOIs).		

**Table 2:** Summary of alignment statistics (minimum, maximum, mean and s.d.) of ChIP- and MeDIP-seq samples aligned with Bowtie2.

**Statistic**	**ChIP-seq**				**MeDIP-seq**			
	**Min**	**Max**	**Average**	**Stdev**	**Min**	**Max**	**Average**	**Stdev**
Mapped Reads (in millions)	14.6	51.0	33.0	6.48	23.9	64.9	33.6	6.40
High-quality reads (in millions)	13.6	47.8	32.1	6.27	21.1	58.1	30.1	5.59
High-quality reads [%]	87.00	100.00	97.32	2.72	82.76	98.36	89.80	4.09
Uniquely mapped reads (in millions)	9.65	41.2	27.9	5.65	15.1	42.6	22.0	4.62
Uniquely mapped reads [%]	60.00	95.00	84.55	8.39	49.92	87.00	65.94	9.70
The table contains the number of mapped reads, number and percentage of high quality uniquely and multi-mapped reads and number and percentage of uniquely mapped reads.								

**Table 3:** Summary of alignment statistics (minimum, maximum, mean and standard deviation) of RNA-seq samples aligned with STAR.

**Statistic**	**RNA-seq**			
	**Min**	**Max**	**Average**	**Stdev**
Mapped reads (in millions)	21.0	61.4	30.2	7.83
Uniquely Mapped Reads (in millions)	14.7	44.3	21.7	5.69
Uniquely Mapped Reads [%]	68.49	73.39	71.62	1.09
Multi-mapped reads (in millions)	6.00	17.1	8.56	2.17
Multi-mapped reads [%]	26.61	31.51	28.38	1.09
The table contains the number of mapped reads, number and percentage of uniquely mapped reads and number and percentage of multi-mapped reads.				
